# Absolute configurations of talaromycones A and B, α-diversonolic ester, and aspergillusone B from endophytic *Talaromyces* sp. ECN211

**DOI:** 10.3762/bjoc.16.28

**Published:** 2020-02-28

**Authors:** Ken-ichi Nakashima, Junko Tomida, Takao Hirai, Yoshiaki Kawamura, Makoto Inoue

**Affiliations:** 1 Laboratory of Medicinal Resources, School of Pharmacy, Aichi Gakuin University, 1-100 Kusumoto-cho, Chikusa-ku, Nagoya, Aichi, Japan; 2 Department of Microbiology, School of Pharmacy, Aichi Gakuin University, 1-100 Kusumoto-cho, Chikusa-ku, Nagoya, Aichi, Japan

**Keywords:** absolute configuration, endophytic fungus, glauconic acid, *Talaromyces*, tetrahydroxanthone, xanthenedione

## Abstract

Talaromycones A (**1**) and B (**2**), new xanthenediones, were isolated from the cultures of *Talaromyces* sp. ECN211, an endophytic fungus, along with α-diversonolic ester (**3**), aspergillusone B (**4**), glauconic acid (**5**), and rosellisin (**6**). The planar structures of **1** and **2** were elucidated by extensive spectroscopic analyses. Furthermore, the absolute configurations of **1**–**4** were determined by single-crystal X-ray diffraction and electronic circular dichroism spectroscopy (ECD). In addition, the crystallographic data for **5** were updated for the first time in over 50 years.

## Introduction

The xanthones, which are a class of phenolic compounds produced by many different organisms, including plants, lichens, fungi, and bacteria, occur as completely aromatized (xanthones in the narrow sense), dihydro, tetrahydro, and hexahydro derivatives [1]. Tetrahydroxanthones produced by fungi are encountered as the monomeric units of multiple dimeric xanthones, including the actinoplanones, albofungins, beticolins, and ergochromes (synonyms: secalonic acids, ergoflavins, and ergochrysins), which are well-known mycotoxins that exhibit toxic, antibacterial, and mutagenic properties [1]. On the other hand, a limited number of monomeric tetrahydroxanthones has been reported as fungal metabolites to date, including the blennolides [2], diversonolic esters [3], and globosuxanthone B [4], among others. Diversonolic esters were firstly reported by Holker from cultures of *Talaromyces diversus*, anamorph *Penicillium diversum* [3]; however, the originally proposed structures of the diversonolic esters were revised after 25 years, following their total synthesis [5]. Furthermore, the compound with the originally proposed structure of β-diversonolic ester had already been isolated as blennolide C from the fungus *Blennoria* sp. prior to structural revision [2]. Consequently, the structures and spectroscopic data of the diversonolic esters and blennolide C were presented. Furthermore, Nicolaou and Li introduced a means by which the absolute configuration can be determined from the total synthesis of α-diversonolic ester (**3**) [5]. However, to the best of our knowledge, data that enabled the determination of the absolute configuration has not been published. Nevertheless, the absolute configuration of aspergillusone B (**4**), which is a compound similar to α-diversonolic ester (**3**), had been determined by comparing the sign of its optical rotation with that of α-diversonolic ester (**3**) [6]. Therefore, the reported absolute configurations of **3** and aspergillusone B (**4**) lack the evidence that supports their assignments, i.e., the absolute configurations of α-diversonolic ester (**3**), aspergillusone B (**4**), and their related compounds are still unknown. As part of our research into compounds produced by endophytic fungi in Japan [7–9], we isolated two new diversonolic ester-related xanthenediones from *Talaromyces* sp. ECN211, namely talaromycones A (**1**) and B (**2**), as well as α-diversonolic ester (**3**) [5], and aspergillusone B (**4**) [6]. Herein, we discuss the determination of the planar structures of the two new compounds **1** and **2** using spectroscopic methodologies, and determination of the absolute configuration of **1**–**4** by single-crystal X-ray diffraction and ECD spectroscopy. In addition, we also updated the crystallographic data for glauconic acid (**5**), a known nonadride.

## Results and Discussion

**Figure 1:**
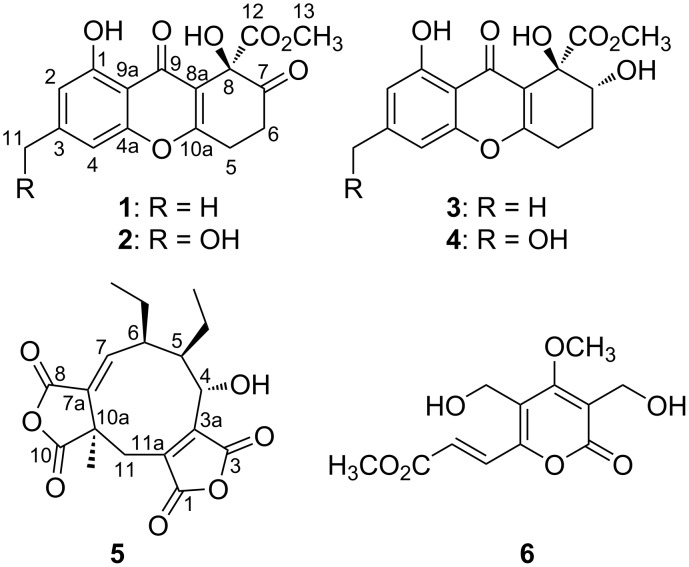
Structures of compounds **1**–**6**.

*Talaromyces* sp. ECN211 was isolated from healthy leaves of *Selaginella tamariscana* and identified by sequencing the D1/D2 26S rRNA gene and the internal transcript spacer (ITS) of its ribosomal DNA [10]. The entire mycelia, which were cultured on 300 plates of 2% malt extract agar (MEA) for 30 d, were extracted three times with MeOH at room temperature and evaporated under reduced pressure to afford the crude extract. The MeOH extract (69.2 g) was then partitioned between ethyl acetate and water. The new compounds **1** and **2** were isolated from the ethyl acetate layer (12.7 g), together with four known compounds (Figure 1), namely α-diversonolic ester (**3**) [5], aspergillusone B (**4**) [6], glauconic acid (**5**) [11–14], and rosellisin (**6**) [15], by repeated silica gel, octadecyl silica (ODS), and Sephadex™ LH-20 column chromatography. The structures of the known compounds were identified on the basis of NMR spectroscopic data from the literature.

Talaromycone A (**1**) was isolated as a colorless solid, and HRESIMS showed a sodium adduct ion peak at *m*/*z* 341.0624 attributable to the molecular formula C_16_H_14_O_7_Na (calcd 341.0637) and indicative of ten indices of hydrogen deficiency. The IR spectrum exhibited absorptions due to hydroxy groups (*ν*_max_ 3408 cm^−1^) and three carbonyl groups (*ν*_max_ 1737, 1709, and 1657 cm^−1^), while the ^1^H NMR spectrum (Table 1) displayed resonances for an aromatic methyl group (δ_H_ 2.42 (3H, s, H_3_-11)), a methyl group adjacent to an oxygen atom (δ_H_ 3.83 (3H, s, H_3_-13)), two pairs of methylene protons (δ_H_ 3.00 (1H, m, H-6), 3.10 (1H, m, H-6), and 3.18 (2H, m, H_2_-5)), two aromatic methine protons (δ_H_ 6.64 (1H, br s, H-2) and 6.72 (1H, br s, H-4)), and a hydrogen-bonded hydroxy group (δ_H_ 11.82 (1H, br s, 4-OH)). The ^13^C NMR (Table 1) and DEPT data showed 16 carbon signals comprising two sp^3^ methyl groups (δ_C_ 22.5, 54.0), two sp^3^ methylene units (δ_C_ 28.0, 34.4), two sp^2^ methine moieties (δ_C_ 107.4, 112.7), and one sp^3^ as well as nine sp^2^ nonprotonated carbon atoms, including three carbonyl carbon atoms (δ_C_ 170.2, 181.8, 200.9). In addition to the presence of four carbon–carbon double bonds and three carbonyl groups, three degrees of unsaturation remained, indicative of a tricyclic ring system in the structure of **1**.

**Table 1:** ^1^H (400 MHz) and ^13^C (100 MHz) NMR data for **1** and **2** in CDCl_3_.

position	talaromycone A (**1**)		talaromycone B (**2**)	
	δ_C_, type	δ_H_, multiplicity	δ_C_, type	δ_H_, multiplicity

1	160.3, C		160.6, C	
2	112.7, CH	6.64, br s	109.3, CH	6.74, br s
3	148.2, C		150.7, C	
4	107.4, CH	6.72, br s	104.3, CH	6.92, br s
4a	155.9, C		156.2, C	
5	28.0, CH_2_	3.18, m	28.1, CH_2_	3.20, m
6	34.4, CH_2_	3.00, m	34.4, CH_2_	3.00, m
		3.10, m		3.10, m
7	200.9, C		200.9, C	
8	75.0, C		75.0, C	
8a	117.2, C		117.4, C	
9	181.8, C		181.0, C	
9a	108.0, C		109.1, C	
10a	165.7, C		166.1, C	
11	22.5, CH_3_	2.42, s	64.2, CH_2_	4.73, s
12	170.2, C		170.1, C	
13	54.0, CH_3_	3.83, s	54.1, CH_3_	3.82, s
4-OH		11.82, br s		11.85, br s

**Figure 2:**
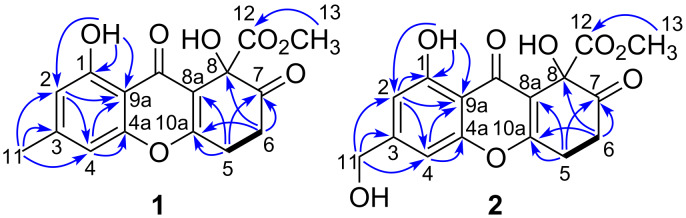
Key HMBC (blue arrows) and COSY (bold bonds) correlations in **1** and **2**.

**Figure 3:**
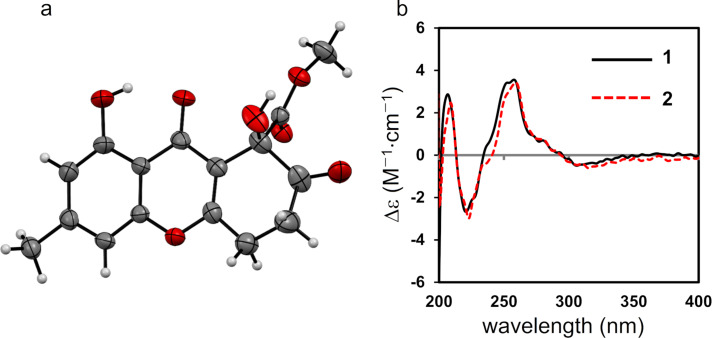
a) ORTEP drawing of **1**, with thermal ellipsoids indicating 50% probability. The atoms of the minor disordered component have been omitted for clarity. b) Electronic circular dichroism spectra of **1** (black solid line) and **2** (red dashed line).

HMBC correlations (Figure 2) from H-2 to C-4 (δ_C_ 107.4) and C-9a (δ_C_ 108.0), from H-4 to C-2 (δ_C_ 112.7), C-4a (δ_C_ 155.9), and C-9a, and from H_3_-11 to C-2 (δ_C_ 112.7), C-3 (δ_C_ 148.2), and C-4 (δ_C_ 107.4) indicated the presence of a tetrasubstituted benzene ring with a methyl group at its C-3 position. The hydrogen-bonded hydroxy group was attached to the C-1 position, as evidenced by HMBC correlations from 4-OH to C-1 (δ_C_ 160.3), C-2 (δ_C_ 112.7), and C-4a (δ_C_ 155.9), which also implied that C-9a was hydrogen-bonded to a carbonyl group. Furthermore, the COSY correlation between H_2_-5 and H_2_-6, along with HMBC correlations from H_2_-5 to C-7 (δ_C_ 200.9), C-8a (δ_C_ 117.2), and C-10a (δ_C_ 165.7) as well as from H_2_-6 to C-7, C-8 (δ_C_ 75.0), and C-10a indicated a six-carbon C-8a/C-10a/C-5/C-6/C-7/C-8 sequence. The remaining methyl proton signal at δ_H_ 3.83 was correlated to a carbonyl carbon signal at δ_C_ 170.2 (C-12), which suggested the presence of a methyl carboxylate group. These substructures, as revealed by 1D and 2D NMR spectroscopic analyses and ^1^H and ^13^C chemical shifts, suggested that **1** was related to the diversonolic esters, but with a carbonyl group at the C-7 position. However, the complete structure of **1** could not be determined by NMR spectroscopy due to a lack of HMBC correlations between the partial structures. Therefore, we crystalized **1** by vapor diffusion with benzene/*n*-hexane. Single-crystal X-ray diffraction using Cu radiation revealed the structure of **1** shown in Figure 3a, although C-5, C-6, C-7, and C-8 were found to be disordered. Furthermore, the absolute configuration at C-8 was determined to be *R* by the Flack parameter (−0.11(17)).

Talaromycone B (**2**) was isolated as a colorless gum, with HRESIMS analysis revealing a sodium adduct ion peak at *m/z* 357.0572, attributable to the molecular formula C_16_H_14_O_8_Na (calcd 357.0586), suggesting that **2** had one more oxygen atom relative to **1**. The ^1^H and ^13^C NMR data for **2** were substantially similar to those of **1**, with the exception that **2** exhibited oxymethylene signals (δ_H_ 4.73 (2H, s); δ_C_ 64.2) instead of the aromatic methyl signals observed for **1** (Table 1). Therefore, C-11 was proposed to be an oxymethylene group, which was also confirmed by HMBC correlations (Figure 2) from the oxymethylene unit to C-2 (δ_C_ 109.3), C-3 (δ_C_ 150.7), and C-4 (δ_C_ 104.3). Since the ECD data of **2** were in good agreement with those of **1** (Figure 3b), the absolute configuration of **2** was also assigned to be *R*.

**Figure 4:**
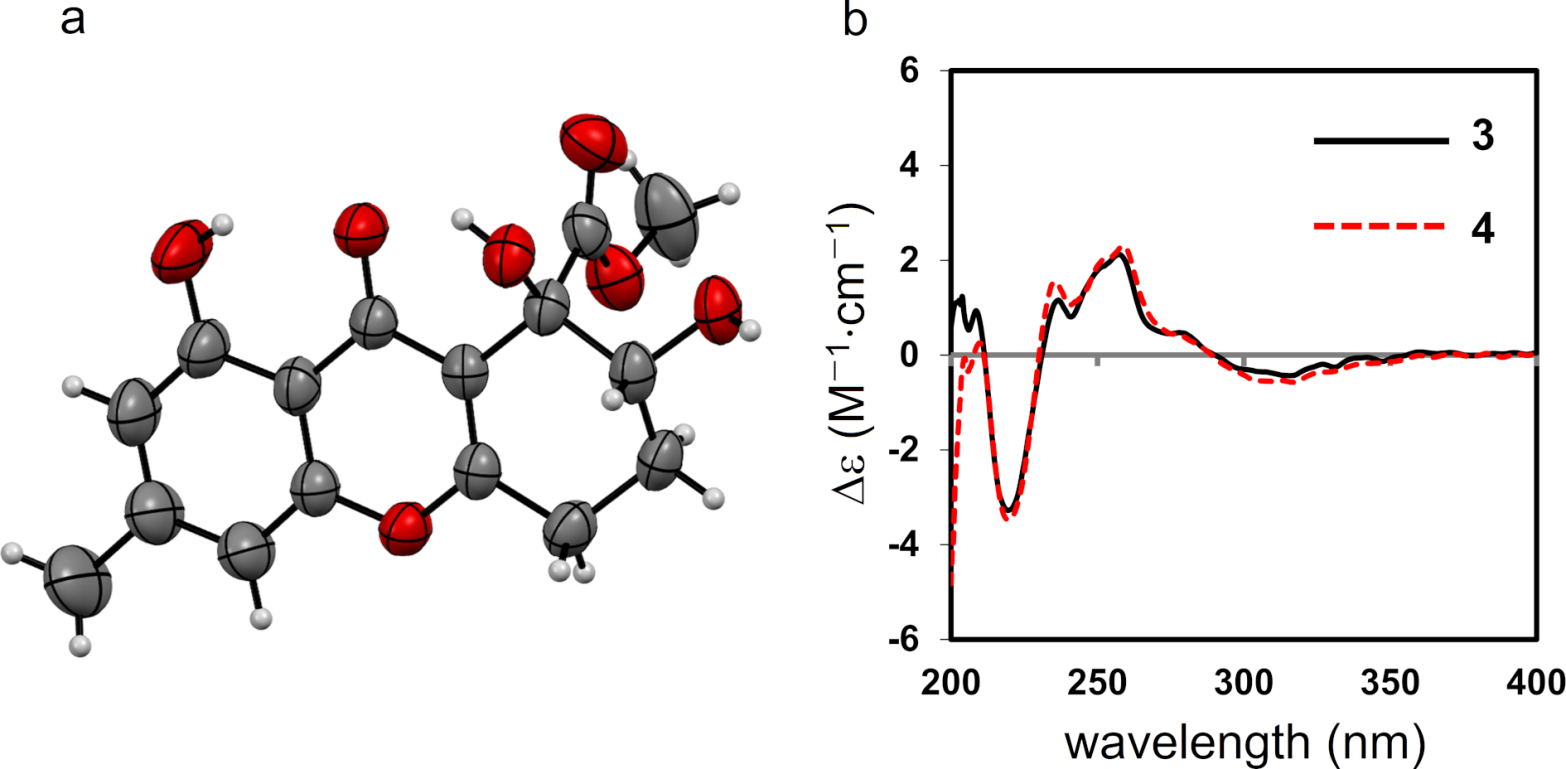
a) ORTEP drawing of **3**, with thermal ellipsoids indicating 50% probability. b) Electronic circular dichroism spectra of **3** (black solid line) and **4** (red dashed line).

Single crystals of α-diversonolic ester (**3**) were obtained as colorless prisms by vapor diffusion with acetone/*n*-hexane, and single-crystal X-ray diffractometry determined the structure shown in Figure 4a. The absolute configurations of the C-7 and C-8 positions were both elucidated to be *R* by the Flack parameter (−0.1(2)). Because the ECD data of aspergillusone B (**4**) were in good agreement with those of **3**, the absolute configuration of **4** was also determined to be *R* in positions 7 and 8 (Figure 4b). This is the first experimental evidence that corroborates the absolute configurations of **3** and **4**, compounds that were reported previously without evidence for this stereochemistry.

**Figure 5:**
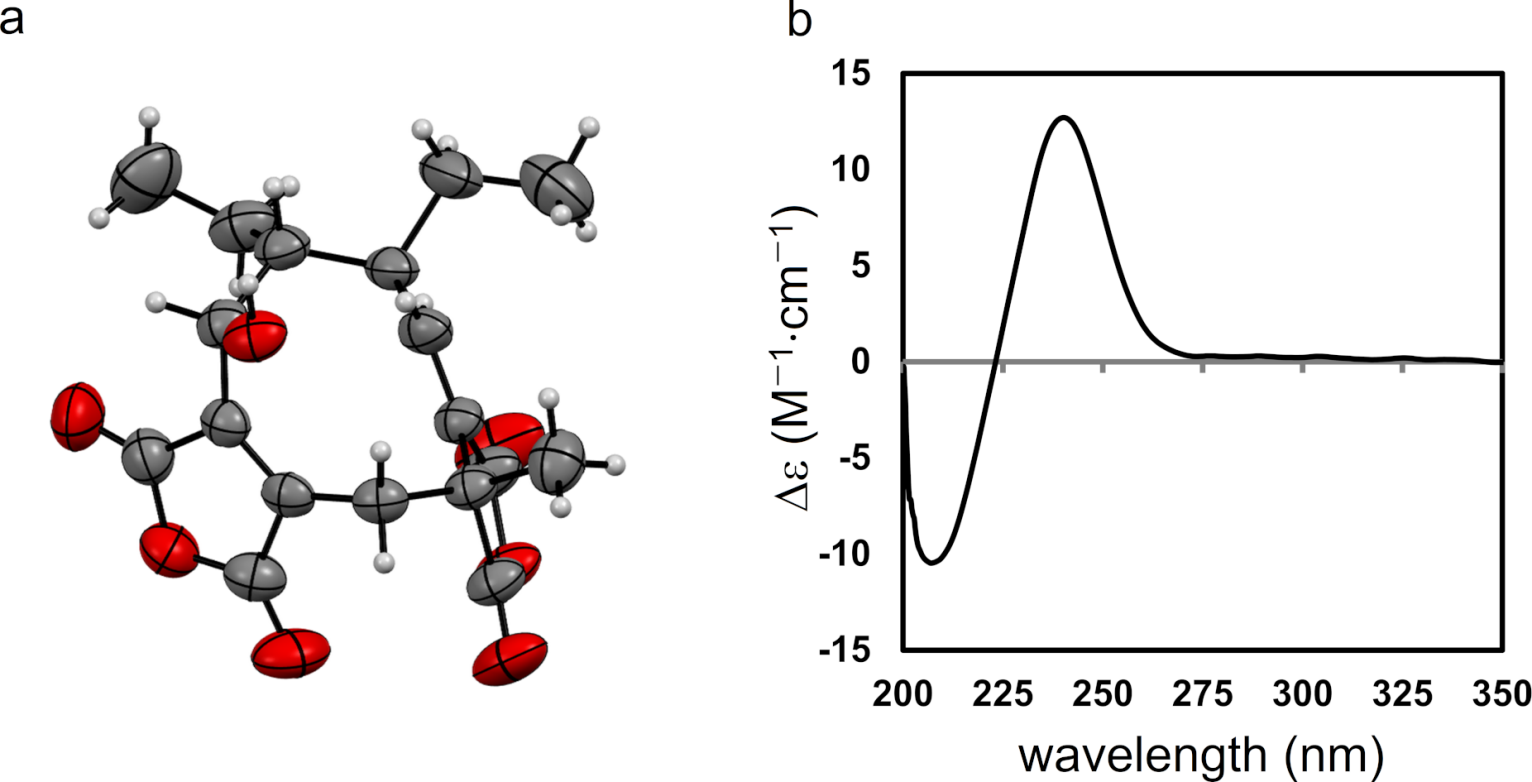
a) ORTEP drawing of **5**, with thermal ellipsoids indicating 50% probability. b) Electronic circular dichroism spectrum of **5**.

We also obtained single crystals of glauconic acid (**5**) as prisms by slow evaporation in CH_2_Cl_2_/*n*-hexane at 10 °C. X-ray diffractometry using Cu radiation revealed the absolute configuration of **5** to be 4*S*,5*R*,6*R*,7*E*,10a*R* by the Flack parameter (−0.13(16), Figure 5a). We also acquired the ECD spectrum of (4*S*,5*R*,6*R*,7*E*,10a*R*)-glauconic acid (Figure 5b). Glauconic acid (**5**) had been originally isolated by Barton et al. in 1965 [11], and its relative configuration had been determined by X-ray diffractometry [16]. In the same year, the absolute configuration of **5** was indirectly established from chemical evidence based on its relative configuration [13]. Meanwhile, no data directly confirming the absolute configuration of **5** has been reported to date. To the best of our knowledge, we present the first X-ray crystallographic data that clearly and directly reveals the absolute configuration of **5**.

## Conclusion

We isolated two new xanthone derivatives, talaromycones A (**1**) and B (**2**), together with two known xanthone derivatives, α-diversonolic ester (**3**) and aspergillusone B (**4**), from the cultures of *Talaromyces* sp. ECN211. The absolute configurations of **3** and **4** had previously only been assigned tentatively and could now be confirmed by single-crystal X-ray diffractometry and ECD spectroscopy. We also revealed the absolute configurations of the new derivatives **1** and **2** using extensive spectroscopic analyses as well as single-crystal X-ray diffractometry. Our crystallization efforts resolved the absolute configuration of α-diversonolic ester (**3**) and its derivatives, which had not been clarified previously.

## Experimental

### General experimental procedures

Optical rotations were measured using a JASCO P-1020 polarimeter, UV spectra were obtained on a Hitachi U-2900 spectrometer, and ECD spectra were acquired on a JASCO J-820 spectropolarimeter. IR spectra were recorded on a Shimadzu FTIR-8400S spectrophotometer, and NMR spectra were acquired on a Jeol JNM-ECZ 400S spectrometer with tetramethylsilane as the internal standard. ESIMS data were obtained using a Shimadzu LCMS-IT-TOF mass spectrometer. Single-crystal X-ray diffraction data were acquired on Rigaku XtaLAB Synergy-S DS and Rigaku R-AXIS RAPID diffractometers using Cu Kα radiation. DNA sequencing was performed with an Applied Biosystems 3130 genetic analyzer. Silica gel AP-300 (Toyota Kako) and Cosmosil 75C 18-OPN (Nacalai Tesque) were used for column chromatography. Silica gel 60 F_254_ and RP-18 F_254S_ (both Merck) were used for TLC.

### Fungal material

The leaves of *Selaginella tamariscana* (Selaginellaceae) were cultivated in Tajimi City, Gifu, Japan. The methods of isolation and identification of endophytic fungi were performed in a similar manner as described previously [8]. Based on the DNA sequencing of ITS of rDNA and the D1/D2 domain of 26S rDNA (Figure S1, Supporting Information File 1), the isolate was found to belong to the genus *Talaromyces*. *Talaromyces* sp. ECN211 exhibited 26S rDNA similarity (95.3%) to *T. trachyspermus* Bhopal (KX66827). The sequence data for *Talaromyces* sp. ECN211 have been deposited at the DNA Data Bank of Japan (DDBJ) under accession numbers LC424445 (ITS) and LC424442 (26S rDNA).

### Fermentation, extraction, and isolation

The methods of fermentation and extraction of the fungus *Talaromyces* sp. ECN211 were performed in a similar manner as described previously [9]. The fungus *Talaromyces* sp. ECN211 was inoculated onto 300 MEA plates without chloramphenicol. After incubation at 27 °C for 30 d, the fermented materials were extracted with MeOH (3 × 8 L) every 24 h at room temperature, and the solution was evaporated in vacuo to afford the MeOH extract (69.2 g). The MeOH extract was partitioned twice with equal amounts of ethyl acetate and water, and the combined ethyl acetate solution was concentrated in vacuo to yield the ethyl acetate-soluble fraction (12.7 g). The ethyl acetate fraction was separated by silica gel column chromatography with CHCl_3_/MeOH (stepwise gradient, 50:1, 30:1, 20:1, 15:1, 10:1, 8:1, and 0:1, v/v) as eluent. The fractions were pooled according to TLC analysis to yield nine combined fractions (F1–9). F2 was subjected to ODS column chromatography with MeCN/H_2_O (stepwise gradient, 3:7, 2:3, and 1:1, v/v) to yield **1** (45.3 mg). F4 was recrystallized from CH_2_Cl_2_ to obtain crude crystals of **5** (1.52 g). The filtrate of F4 was separated by silica gel column chromatography with *n*-hexane/acetone (stepwise gradient, 3:1 and 2:1, v/v) to yield **3** (434.1 mg). F5 was purified by silica gel column chromatography with *n*-hexane/acetone (5:2) to yield **2** (31.7 mg). The CH_2_Cl_2_-soluble part of F8 was separated by silica gel column chromatography with *n*-hexane/acetone (2:1, v/v) to yield **4** (34.3 mg) and **6** (58.2 mg).

### Talaromycone A (**1**)

Colorless plates (benzene/*n*-hexane). mp 176–179 °C; [α]_D_^25^ +18.4 (*c* 0.1, MeOH); ^1^H and ^13^C NMR see Table 1; UV (MeOH) λ_max_ (log ε) 327 (3.68), 260 (4.24), 240 (4.39), 229 nm (4.35); IR (KBr) ν_max_: 3408, 1737, 1709, 1657, 1620, 1599, 1498, 1452, 1352, 1288 cm^−1^; HRESIMS (*m*/*z*): [M + Na]^+^ calcd for C_16_H_14_O_7_Na, 341.0637; found, 341.0624.

### Talaromycone B (**2**)

Colorless gum. [α]_D_^25^ +18.0 (*c* 0.1, MeOH); ^1^H and ^13^C NMR see Table 1; UV (MeOH) λ_max_ (log ε) 328 (3.61), 263 (4.07), 239 (4.32), 228 nm (4.26); IR (KBr) ν_max_: 3437, 1751, 1734, 1654, 1620, 1491, 1448, 1290, 1271, 1205 cm^−1^; HRESIMS (*m*/*z*): [M + Na]^+^ calcd for C_16_H_14_O_7_Na, 357.0586; found, 357.0572.

### α-Diversonolic ester (**3**)

Colorless plates (acetone/*n*-hexane). mp 206–209 °C; [α]_D_^25^ +23.2 (*c* 0.1, MeOH).

### Aspergillusone (**4**)

Colorless gum. [α]_D_^25^ +31.8 (*c* 0.1, MeOH).

### Glauconic acid (**5**)

Colorless prism (CH_2_Cl_2_/*n*-hexane). mp 215–218 °C; [α]_D_^25^ +56.0 (*c* 0.1, MeOH).

### X-ray diffraction data for **1**

C_16_H_14_O_7_, *M* = 318.27, crystal size 0.22 × 0.04 × 0.005 mm^3^, monoclinic, space group *C*2, *a* = 13.8091(5) Å, *b* = 5.1557(2) Å, *c* = 20.4560(7) Å, *V* = 1419.64(9) Å^3^, *Z* = 4, α = γ = 90°, β = 102.896°, ρ(calcd) = 1.489 g·cm^−3^, F(000) = 664, reflections collected/unique 2435/250 (*R*(int) = 0.0405), final *R* indices (*I >* 2σ (*I*)) *R_1_* = 0.0678, *wR_2_* = 0.1977, goodness of fit = 1.070, Flack parameter = −0.11(17). Crystallographic data for **1** have been deposited with the Cambridge Crystallographic Data Centre (CCDC 1952664). The data can be obtained free of charge from the Cambridge Crystallographic Data Centre via http://www.ccdc.cam.ac.uk/data_request/cif.

### X-ray diffraction data for **3**

C_16_H_16_O_7_, *M* = 320.30, crystal size 0.30 × 0.10 × 0.05 mm^3^, monoclinic, space group *P*2_1_, *a* = 8.7229(4) Å, *b* = 8.3549(3) Å, *c* = 21.0702(15) Å, *V* = 1503.53(14) Å^3^, *Z* = 4, α = γ = 90°, β = 101.725°, ρ(calcd) = 1.415 g·cm^−3^, F(000) = 672, reflections collected/unique 16755/4850 (*R*(int) = 0.1218), final *R* indices (*I >* 2σ (*I*)) *R_1_* = 0.0618, *wR_2_* = 0.1720, goodness of fit = 1.005, Flack parameter = −0.1(2). Crystallographic data for **5** have been deposited with the Cambridge Crystallographic Data Centre (CCDC 1959520).

### X-ray diffraction data for **5**

C_18_H_20_O_7_, *M* = 348.35, crystal size 0.26 × 0.20 × 0.14 mm^3^, monoclinic, space group *P*2_1_, *a* = 7.3325(3) Å, *b* = 13.9964(5) Å, *c* = 9.0451(3) Å, *V* = 863.28(7) Å^3^, *Z* = 2, α = γ = 90°, β = 111.57°, ρ(calcd) = 1.340 g·cm^−3^, F(000) = 368, reflections collected/unique 9852/2994 (*R*(int) = 0.0707), final *R* indices (*I >* 2σ (*I*)) *R_1_* = 0.0561, *wR_2_* = 0.1458, goodness of fit = 1.084, Flack parameter = −0.13(16). Crystallographic data for **5** have been deposited with the Cambridge Crystallographic Data Centre (CCDC 1952663).

## Supporting Information

File 1 A phytogenic tree for ECN211 and related species and NMR spectra of **1** and **2**.

File 2 Crystal structure information files for **1, 3**, and **5**.
