# Supplementary material for: Absolute configurations of talaromycones A and B, α-diversonolic ester, and aspergillusone B from endophytic Talaromyces sp. ECN211
Source: Beilstein J Org Chem. 2020 Feb 28;16:290–6. doi: 10.3762/bjoc.16.28 (PMC7059540; doi:10.3762/bjoc.16.28)
Supplement: File 1 — A phytogenic tree for ECN211 and related species and NMR spectra of 1 and 2. [file Beilstein_J_Org_Chem-16-290-s001.pdf]

BEILSTEIN JOURNAL OF ORGANIC CHEMISTRY

Supporting Information

for

Absolute configurations of talaromycones A and B, α-diversonolic ester, and aspergillusone B from endophytic *Talaromyces* sp. ECN211

Ken-ichi Nakashima, Junko Tomida, Takao Hirai, Yoshiaki Kawamura and Makoto Inoue

Beilstein J. Org. Chem. **2020,** *16,* 290–296. doi:10.3762/bjoc.16.28

A phytogenic tree for ECN211 and related species and NMR spectra of 1 and 2

License and Terms: This is a supporting information file under the terms of the Creative Commons Attribution License (https://creativecommons.org/licenses/by/4.0). Please note that the reuse, redistribution and reproduction in particular requires that the authors and source are credited.
The license is subject to the *Beilstein Journal of Organic Chemistry* terms and conditions: (https://www.beilstein-journals.org/bjoc)

Table of Contents

Figure S1: A phytogenic tree for ECN211 and related species based on the D1/D2 domain of 26SrDNA sequences.

Figure S2: ^{1}H NMR spectrum (400 MHz, $CDCl_3$) of **1**

Figure S3: ^{13}C NMR and DEPT-135 spectra (100 MHz, $CDCl_3$) of **1**

Figure S4: HMQC spectrum ($CDCl_3$) of **1**

Figure S5: HMBC spectrum ($CDCl_3$) of **1**

Figure S6: ^{1}H NMR spectrum (400 MHz, $CDCl_3$) of **2**

Figure S7: ^{13}C NMR and DEPT-135 spectra (100 MHz, $CDCl_3$) of **2**

Figure S8: HMQC spectrum ($CDCl_3$) of **2**

Figure S9: HMBC spectrum ($CDCl_3$) of **2**

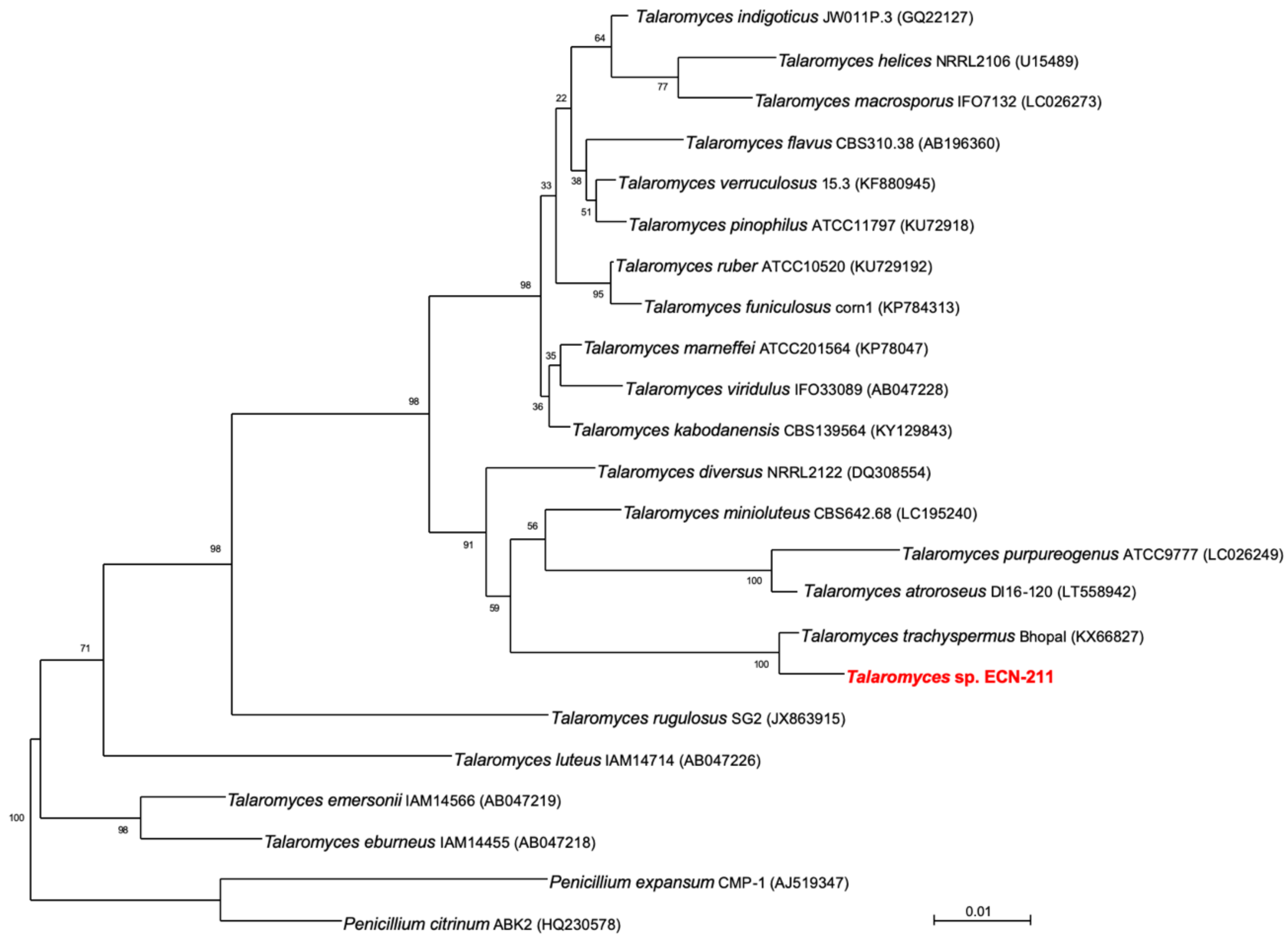

Figure S1: A phytogenic tree for ECN211 and related species based on the D1/D2 domain of 26SrDNA sequences.

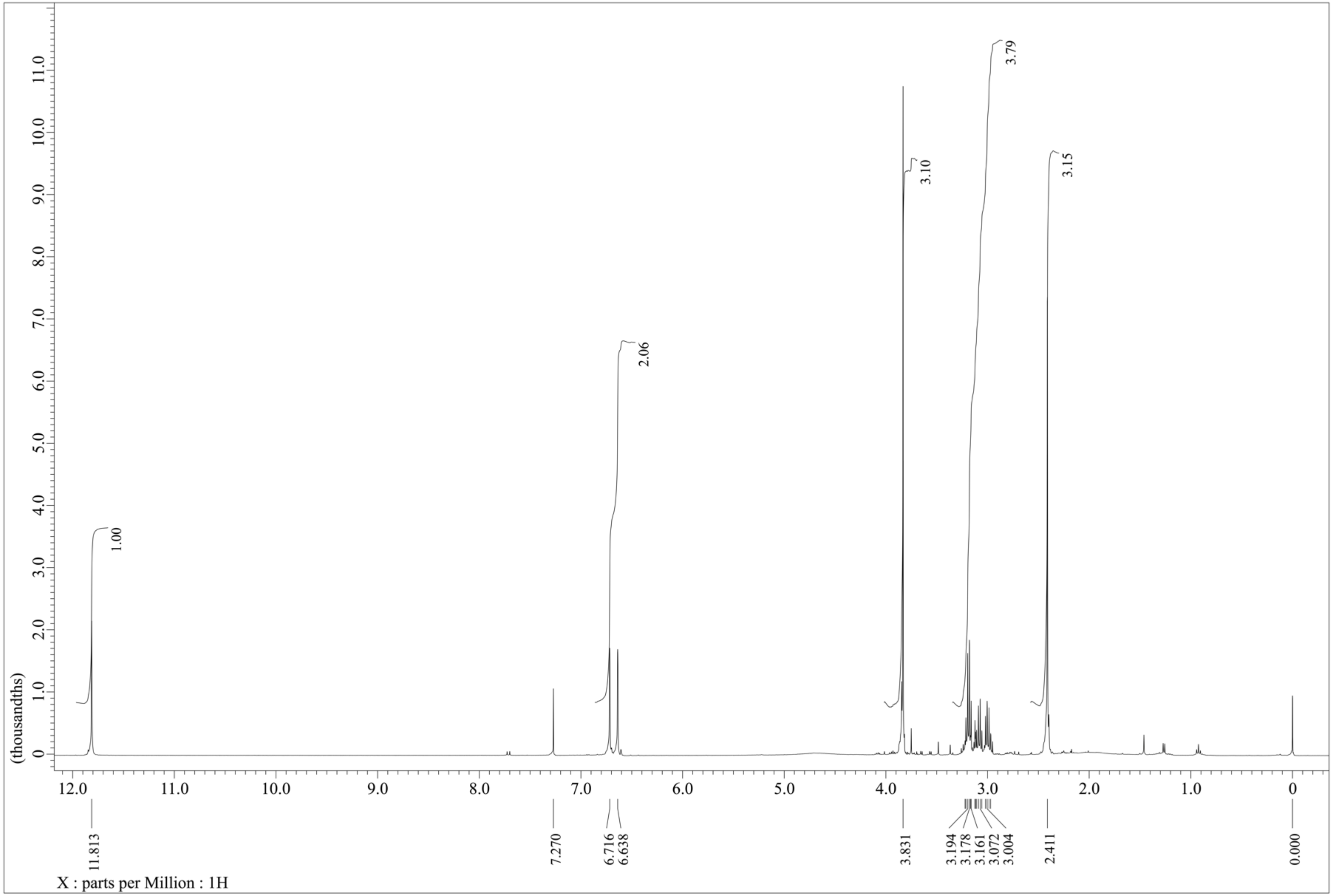

Figure S2: ^{1}H NMR spectrum (400 MHz, $CDCl_3$) of **1**.

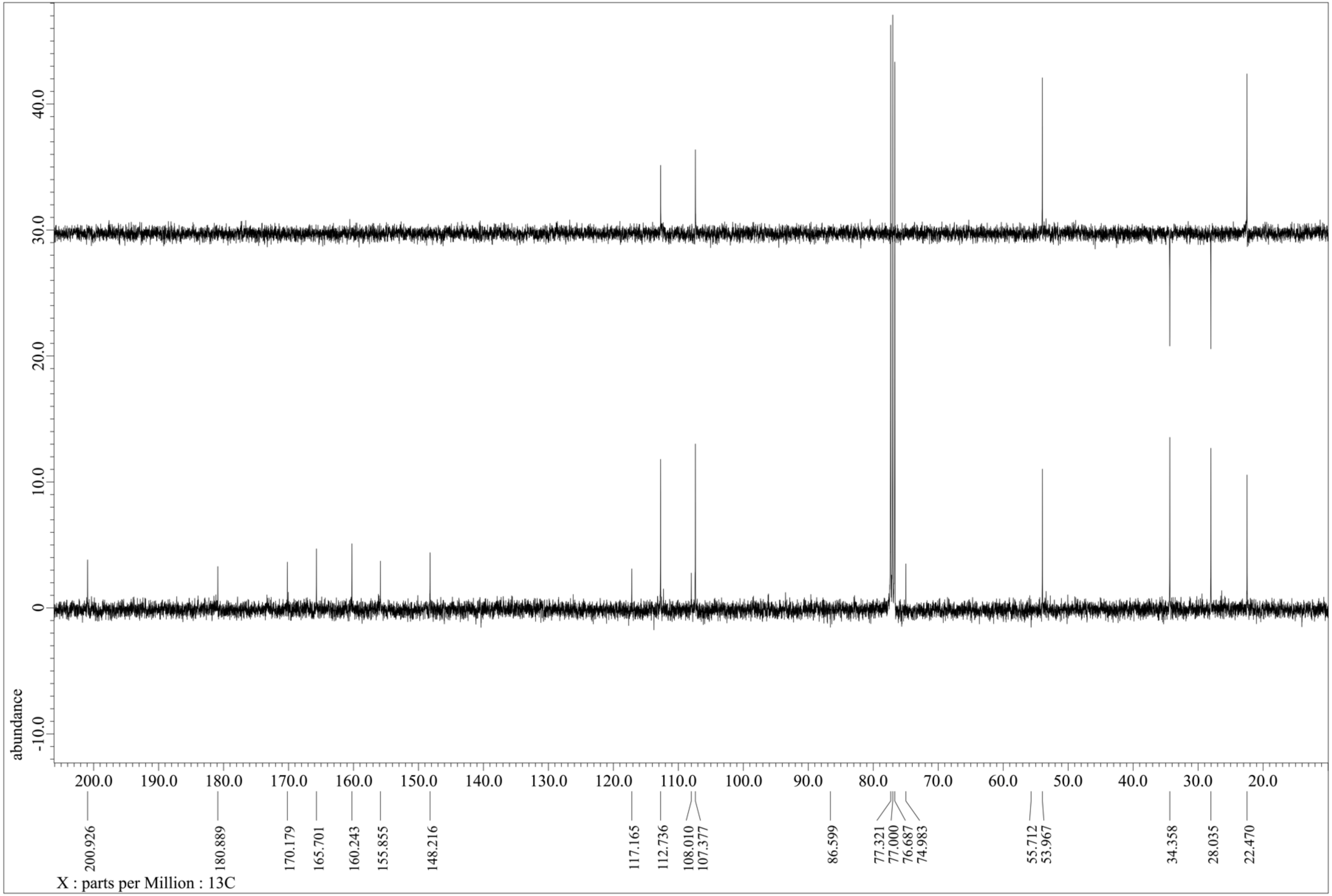

Figure S3: ^{13}C NMR and DEPT-135 spectra (100 MHz, $CDCl_3$) of **1**.

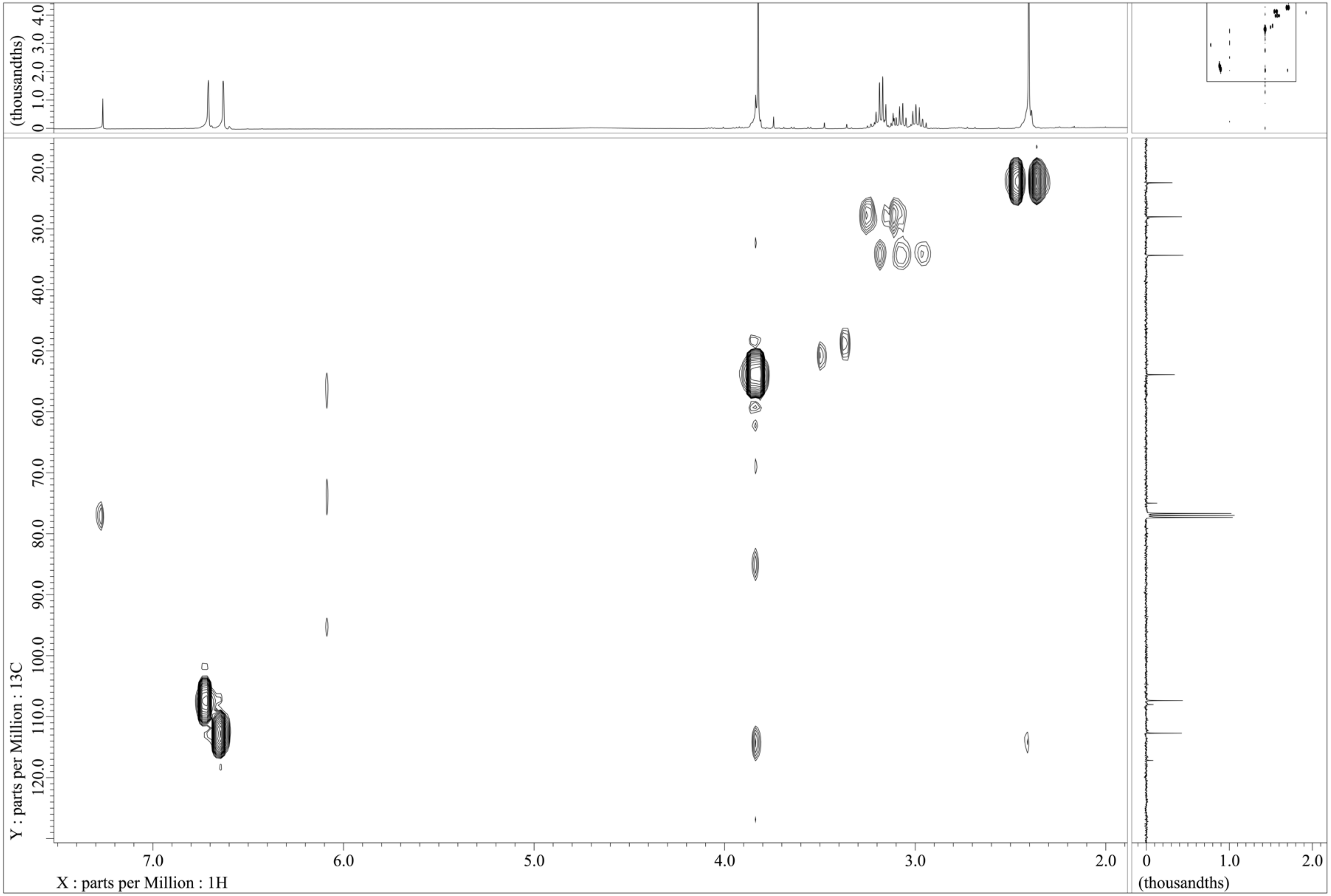

Figure S4: HMQC spectrum ($CDCl_3$) of **1**.

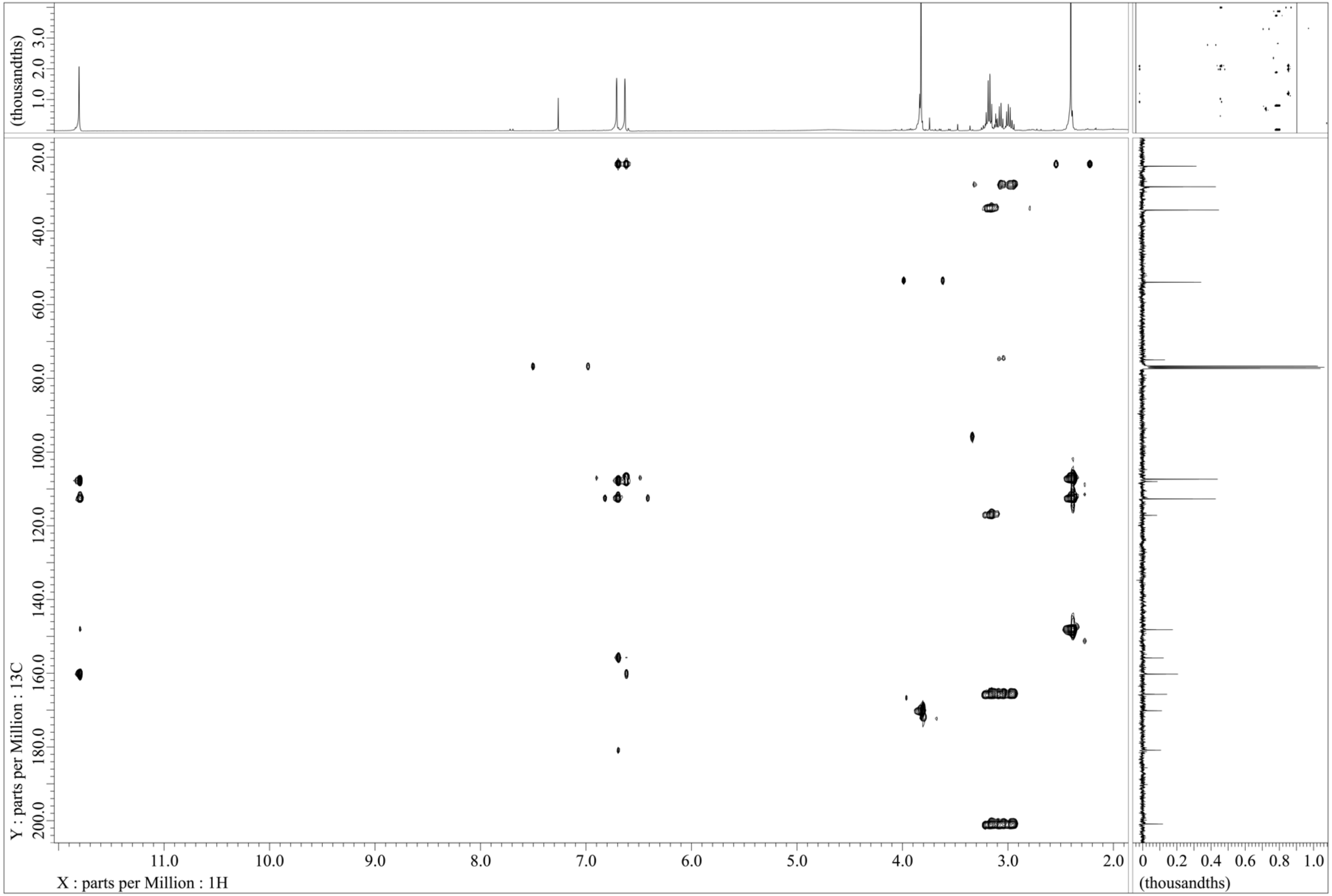

Figure S5: HMBC spectrum ($CDCl_3$) of **1**.

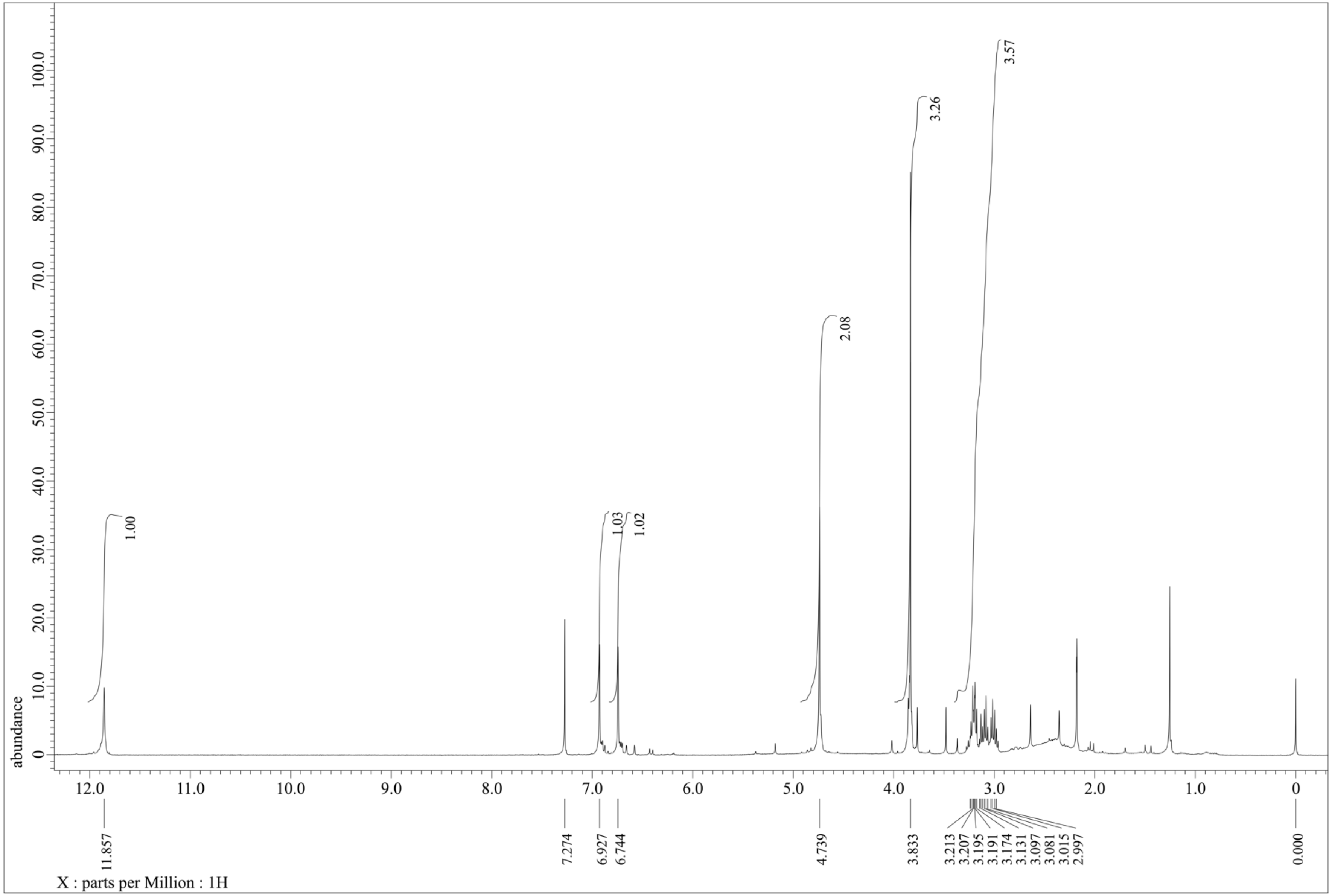

Figure S6: ^{1}H NMR spectrum (400 MHz, $CDCl_3$) of **2**.

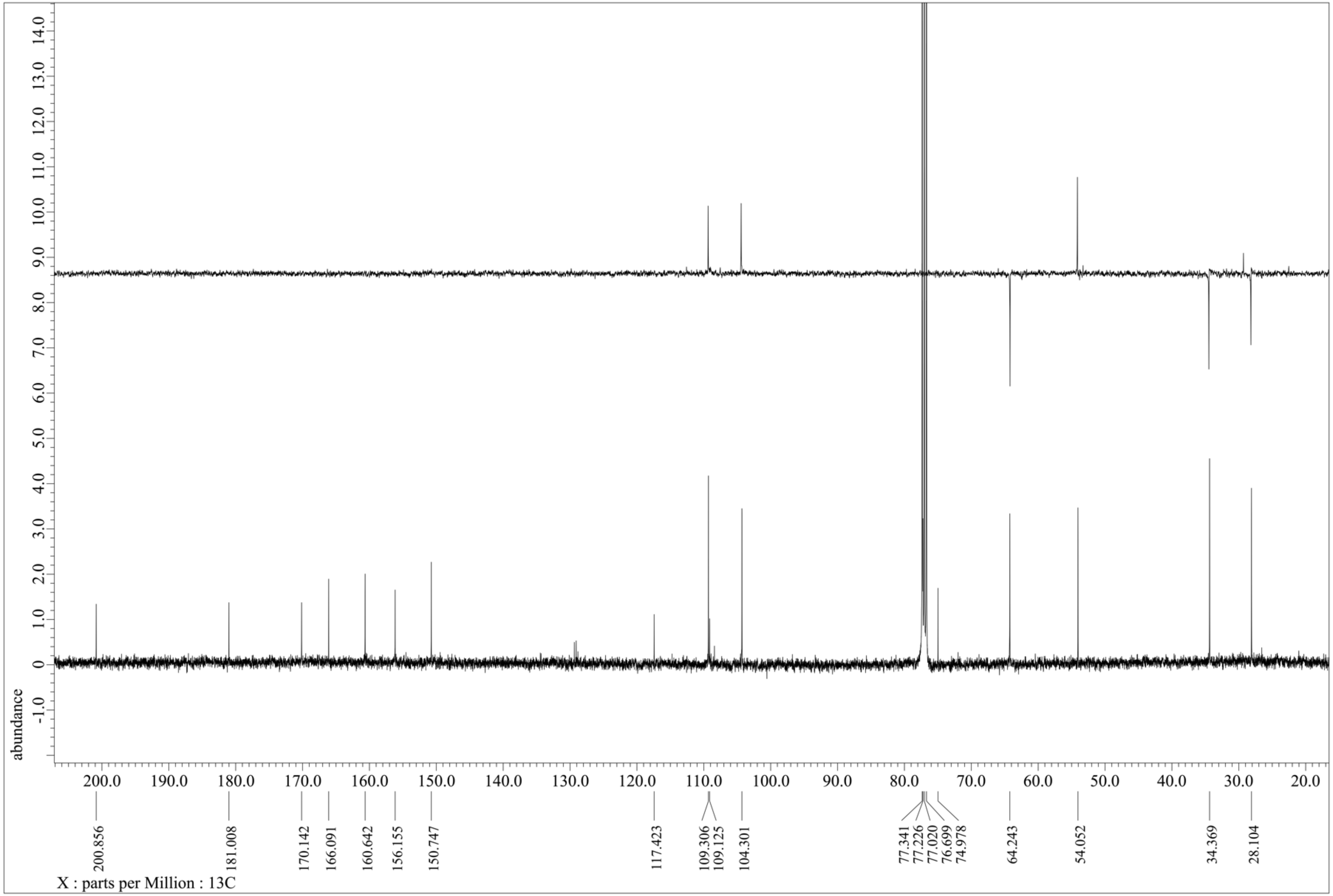

Figure S7: ^{13}C NMR and DEPT-135 spectra (100 MHz, $CDCl_3$) of **2**.

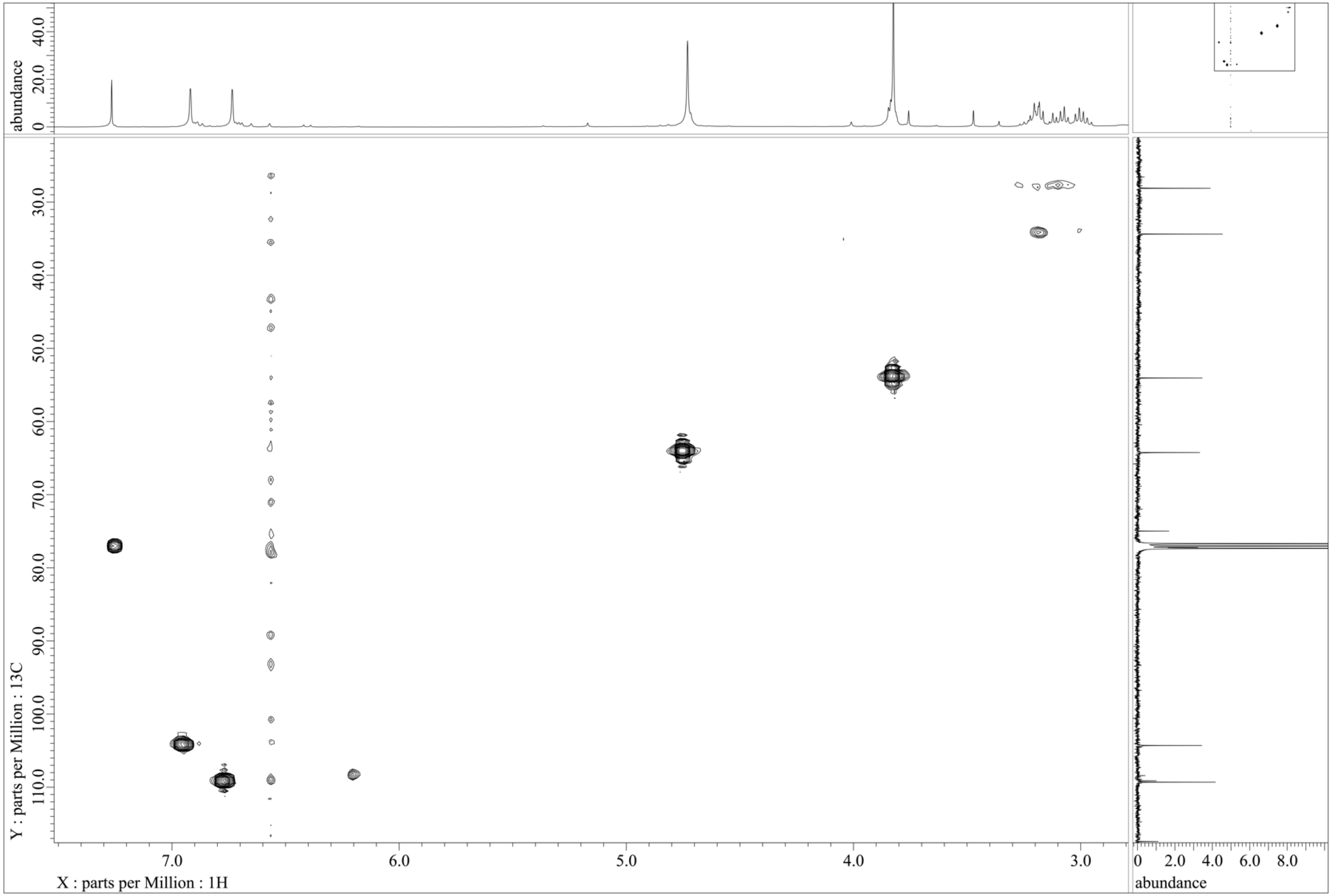

Figure S8: HMQC spectrum ($CDCl_3$) of **2**.

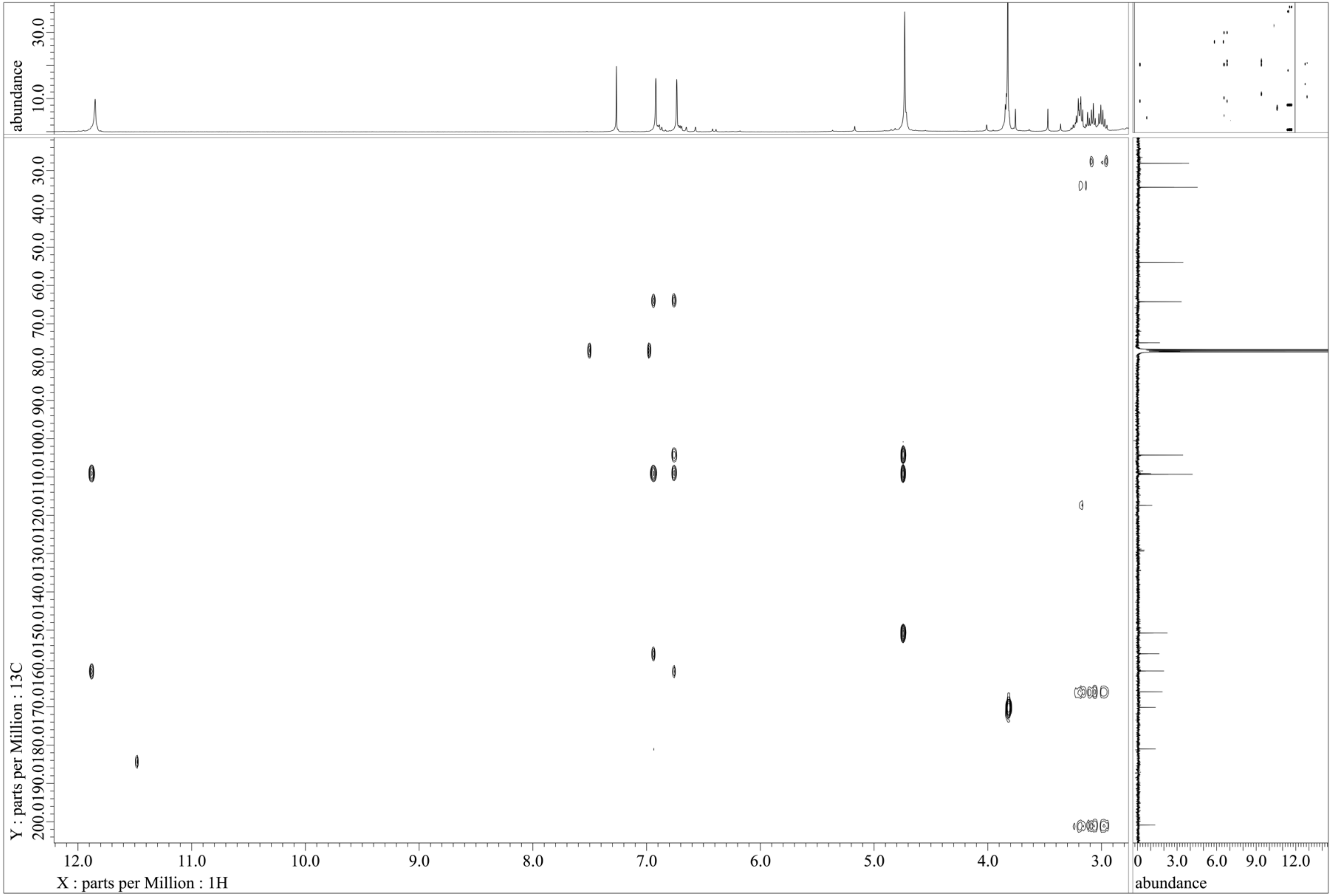

Figure S9: HMBC spectrum ($CDCl_3$) of **2**.
