# Supplementary material for: Absolute configurations of talaromycones A and B, α-diversonolic ester, and aspergillusone B from endophytic Talaromyces sp. ECN211
Source: Beilstein J Org Chem. 2020 Feb 28;16:290–6. doi: 10.3762/bjoc.16.28 (PMC7059540; doi:10.3762/bjoc.16.28)

checkCIF/PLATON report

You have not supplied any structure factors. As a result the full set of tests cannot be run.

THIS REPORT IS FOR GUIDANCE ONLY. IF USED AS PART OF A REVIEW PROCEDURE FOR PUBLICATION, IT SHOULD NOT REPLACE THE EXPERTISE OF AN EXPERIENCED CRYSTALLOGRAPHIC REFEREE.

No syntax errors found.   CIF dictionary   Interpreting this report

Datablock: 514

```
Bond precision:  C-C = 0.0074 A          Wavelength=1.54187

Cell:            a=8.7229(4)    b=8.3549(3)         c=21.0702(15)
                 alpha=90       beta=101.725(3)     gamma=90
Temperature:     296 K

                Calculated                 Reported
Volume          1503.53(14)                1503.53(14)
Space group     P 21                       P 1 21 1
Hall group      P 2yb                      P 2yb
Moiety formula  C16 H16 O7                 C16 H16 O7
Sum formula     C16 H16 O7                 C16 H16 O7
Mr              320.29                     320.30
Dx,g cm-3       1.415                      1.415
Z               4                          4
Mu (mm-1)       0.952                      0.955
F000            672.0                      672.0
F000'           674.47
h,k,lmax        10,10,25                   10,9,25
Nref            5483[ 2947]                4850
Tmin,Tmax       0.892,0.953
Tmin'           0.751

Correction method= Not given

Data completeness= 1.65/0.88      Theta(max)= 67.978

R(reflections)= 0.0618( 4414)     wR2(reflections)= 0.1720( 4850)

S = 1.005                  Npar= 425
```

The following ALERTS were generated. Each ALERT has the format
test-name_ALERT_alert-type_alert-level.
Click on the hyperlinks for more details of the test.

Alert level C

```
RINTA01_ALERT_3_C  The value of Rint is greater than 0.12
            Rint given   0.122
PLAT089_ALERT_3_C Poor Data / Parameter Ratio (Zmax < 18) ........       6.93 Note
PLAT213_ALERT_2_C Atom O207            has ADP max/min Ratio .....        3.6 prolat
PLAT220_ALERT_2_C Non-Solvent  Resd 2  C   Ueq(max)/Ueq(min) Range        3.3 Ratio
PLAT220_ALERT_2_C Non-Solvent  Resd 2  O   Ueq(max)/Ueq(min) Range        3.4 Ratio
PLAT234_ALERT_4_C Large Hirshfeld Difference C213     --C214     .       0.19 Ang.
PLAT242_ALERT_2_C Low    'MainMol' Ueq as Compared to Neighbors of       C213 Check
PLAT250_ALERT_2_C Large U3/U1 Ratio for Average U(i,j) Tensor ....        2.3 Note
PLAT260_ALERT_2_C Large Average Ueq of Residue Including      O201      0.106 Check
PLAT340_ALERT_3_C Low Bond Precision on  C-C Bonds ...............    0.00737 Ang.
```

Alert level G

```
CHEMS02_ALERT_1_G  Please check that you have entered the correct
            _publ_requested_category classification of your compound;
            FI or CI or EI for inorganic; FM or CM or EM for metal-organic;
            FO or CO or EO for organic.
            From the CIF: _publ_requested_category   CHOOSE FI FM FO CI CM CO or A
            From the CIF: _chemical_formula_sum :C16 H16 O7
PLAT007_ALERT_5_G Number of Unrefined Donor-H Atoms ..............          6 Report
PLAT020_ALERT_3_G The Value of Rint is Greater Than 0.12 .........      0.122 Report
PLAT072_ALERT_2_G SHELXL First  Parameter in WGHT  Unusually Large       0.11 Report
PLAT791_ALERT_4_G Model has Chirality at C103        (Chiral SPGR)          R Verify
PLAT791_ALERT_4_G Model has Chirality at C104        (Chiral SPGR)          R Verify
PLAT791_ALERT_4_G Model has Chirality at C203        (Chiral SPGR)          R Verify
PLAT791_ALERT_4_G Model has Chirality at C204        (Chiral SPGR)          R Verify
```

---

0 **ALERT level A** = Most likely a serious problem - resolve or explain
0 **ALERT level B** = A potentially serious problem, consider carefully
10 **ALERT level C** = Check. Ensure it is not caused by an omission or oversight
8 **ALERT level G** = General information/check it is not something unexpected

1 ALERT type 1 CIF construction/syntax error, inconsistent or missing data
7 ALERT type 2 Indicator that the structure model may be wrong or deficient
4 ALERT type 3 Indicator that the structure quality may be low
5 ALERT type 4 Improvement, methodology, query or suggestion
1 ALERT type 5 Informative message, check

It is advisable to attempt to resolve as many as possible of the alerts in all categories. Often the minor alerts point to easily fixed oversights, errors and omissions in your CIF or refinement strategy, so attention to these fine details can be worthwhile. In order to resolve some of the more serious problems it may be necessary to carry out additional measurements or structure refinements. However, the purpose of your study may justify the reported deviations and the more serious of these should normally be commented upon in the discussion or experimental section of a paper or in the "special_details" fields of the CIF. checkCIF was carefully designed to identify outliers and unusual parameters, but every test has its limitations and alerts that are not important in a particular case may appear. Conversely, the absence of alerts does not guarantee there are no aspects of the results needing attention. It is up to the individual to critically assess their own results and, if necessary, seek expert advice.

Publication of your CIF in IUCr journals

A basic structural check has been run on your CIF. These basic checks will be run on all CIFs submitted for publication in IUCr journals (*Acta Crystallographica*, *Journal of Applied Crystallography*, *Journal of Synchrotron Radiation*); however, if you intend to submit to *Acta Crystallographica Section C* or *E* or *IUCrData*, you should make sure that full publication checks are run on the final version of your CIF prior to submission.

Publication of your CIF in other journals

Please refer to the *Notes for Authors* of the relevant journal for any special instructions relating to CIF submission.

PLATON version of 07/08/2019; check.def file version of 30/07/2019

Datablock 514 - ellipsoid plot

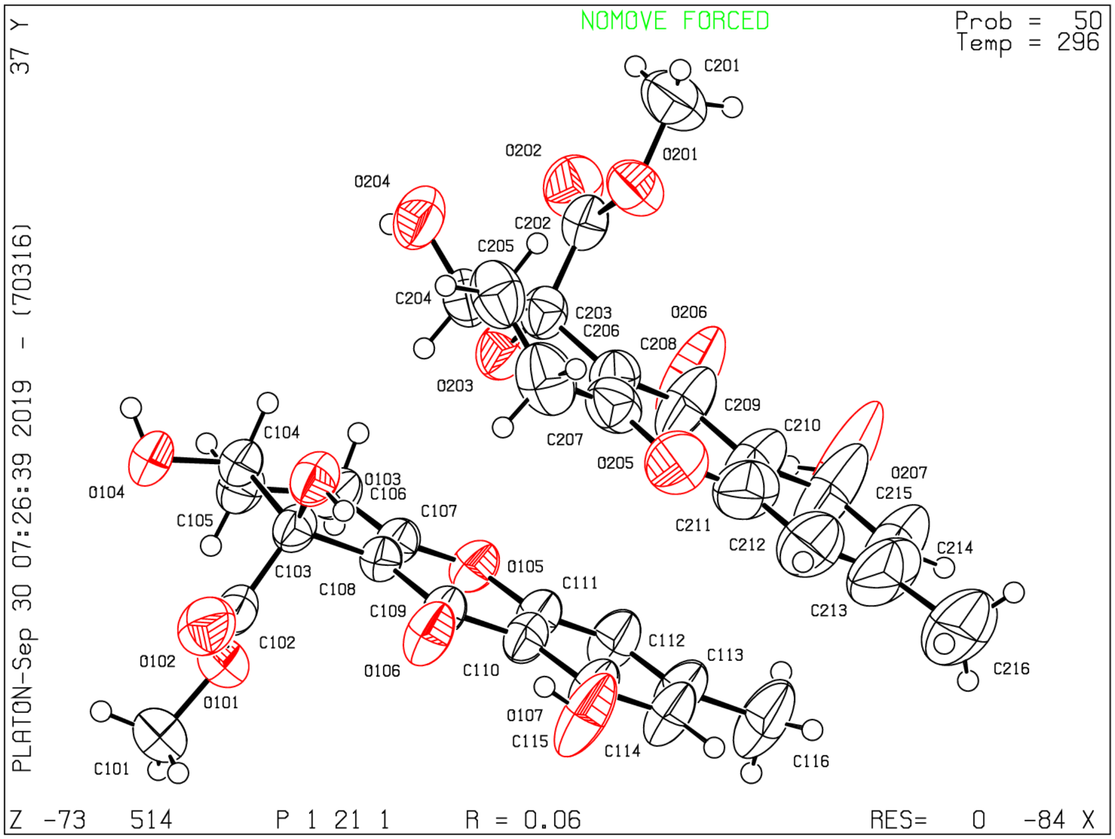

Supplement: File 2 — Crystal structure information files for 1, 3, and 5. [file Beilstein_J_Org_Chem-16-290-s002.zip › Crystal files/checkcif3.pdf]
