# Supplementary material for: Absolute configurations of talaromycones A and B, α-diversonolic ester, and aspergillusone B from endophytic Talaromyces sp. ECN211
Source: Beilstein J Org Chem. 2020 Feb 28;16:290–6. doi: 10.3762/bjoc.16.28 (PMC7059540; doi:10.3762/bjoc.16.28)

checkCIF/PLATON report

You have not supplied any structure factors. As a result the full set of tests cannot be run.

THIS REPORT IS FOR GUIDANCE ONLY. IF USED AS PART OF A REVIEW PROCEDURE FOR PUBLICATION, IT SHOULD NOT REPLACE THE EXPERTISE OF AN EXPERIENCED CRYSTALLOGRAPHIC REFEREE.

No syntax errors found.   CIF dictionary   Interpreting this report

Datablock: exp_1004

```
Bond precision:  C-C = 0.0078 A          Wavelength=1.54184

Cell:           a=13.8091(5)    b=5.1557(2)         c=20.4560(7)
                alpha=90        beta=102.896(3)     gamma=90
Temperature:    100 K

                Calculated                Reported
Volume          1419.64(9)                1419.64(9)
Space group     C 2                       C 1 2 1
Hall group      C 2y                      C 2y
Moiety formula  C16 H14 O7                C16 H14 O7
Sum formula     C16 H14 O7                C16 H14 O7
Mr              318.27                    318.27
Dx,g cm-3       1.489                     1.489
Z               4                         4
Mu (mm-1)       1.007                     1.007
F000            664.0                     664.0
F000'           666.47
h,k,lmax        16,6,24                   16,6,24
Nref            2599[ 1457]               2435
Tmin,Tmax       0.953,0.995               0.601,1.000
Tmin'           0.801

Correction method= # Reported T Limits: Tmin=0.601 Tmax=1.000
AbsCorr = MULTI-SCAN

Data completeness= 1.67/0.94      Theta(max)= 68.180

R(reflections)= 0.0678( 2215)     wR2(reflections)= 0.1977( 2435)

S = 1.070                  Npar= 250
```

The following ALERTS were generated. Each ALERT has the format
test-name_ALERT_alert-type_alert-level.
Click on the hyperlinks for more details of the test.

Alert level C

```
DIFMX02_ALERT_1_C  The maximum difference density is > 0.1*ZMAX*0.75
            The relevant atom site should be identified.
PLAT089_ALERT_3_C Poor Data / Parameter Ratio (Zmax < 18) ........       5.83 Note
PLAT097_ALERT_2_C Large Reported Max.  (Positive) Residual Density       0.75 eA-3
PLAT213_ALERT_2_C Atom O0              has ADP max/min Ratio .....        4.0 prolat
PLAT234_ALERT_4_C Large Hirshfeld Difference C00O     --C00L     .       0.17 Ang.
PLAT340_ALERT_3_C Low Bond Precision on  C-C Bonds ...............    0.00777 Ang.
```

Alert level G

```
PLAT002_ALERT_2_G Number of Distance or Angle Restraints on AtSite         10 Note
PLAT003_ALERT_2_G Number of Uiso or Uij Restrained non-H Atoms ...         10 Report
PLAT007_ALERT_5_G Number of Unrefined Donor-H Atoms ..............          3 Report
PLAT072_ALERT_2_G SHELXL First  Parameter in WGHT  Unusually Large       0.14 Report
PLAT176_ALERT_4_G The CIF-Embedded .res File Contains SADI Records          9 Report
PLAT178_ALERT_4_G The CIF-Embedded .res File Contains SIMU Records          2 Report
PLAT187_ALERT_4_G The CIF-Embedded .res File Contains RIGU Records          2 Report
PLAT301_ALERT_3_G Main Residue  Disorder ..............(Resd  1  )        17% Note
PLAT432_ALERT_2_G Short Inter X...Y Contact  O006     ..C00F             2.97 Ang.
                                                   x,-1+y,z  =      1_545 Check
PLAT720_ALERT_4_G Number of Unusual/Non-Standard Labels ..........         46 Note
PLAT860_ALERT_3_G Number of Least-Squares Restraints .............        172 Note
PLAT933_ALERT_2_G Number of OMIT Records in Embedded .res File ...          3 Note
```

   0 **ALERT level A** = Most likely a serious problem - resolve or explain
   0 **ALERT level B** = A potentially serious problem, consider carefully
   6 **ALERT level C** = Check. Ensure it is not caused by an omission or oversight
  12 **ALERT level G** = General information/check it is not something unexpected

   1 ALERT type 1 CIF construction/syntax error, inconsistent or missing data
   7 ALERT type 2 Indicator that the structure model may be wrong or deficient
   4 ALERT type 3 Indicator that the structure quality may be low
   5 ALERT type 4 Improvement, methodology, query or suggestion
   1 ALERT type 5 Informative message, check

It is advisable to attempt to resolve as many as possible of the alerts in all categories. Often the minor alerts point to easily fixed oversights, errors and omissions in your CIF or refinement strategy, so attention to these fine details can be worthwhile. In order to resolve some of the more serious problems it may be necessary to carry out additional measurements or structure refinements. However, the purpose of your study may justify the reported deviations and the more serious of these should normally be commented upon in the discussion or experimental section of a paper or in the "special_details" fields of the CIF. checkCIF was carefully designed to identify outliers and unusual parameters, but every test has its limitations and alerts that are not important in a particular case may appear. Conversely, the absence of alerts does not guarantee there are no aspects of the results needing attention. It is up to the individual to critically assess their own results and, if necessary, seek expert advice.

Publication of your CIF in IUCr journals

A basic structural check has been run on your CIF. These basic checks will be run on all CIFs submitted for publication in IUCr journals (*Acta Crystallographica*, *Journal of Applied Crystallography*, *Journal of Synchrotron Radiation*); however, if you intend to submit to *Acta Crystallographica Section C* or *E* or *IUCrData*, you should make sure that full publication checks are run on the final version of your CIF prior to submission.

Publication of your CIF in other journals

Please refer to the *Notes for Authors* of the relevant journal for any special instructions relating to CIF submission.

PLATON version of 07/08/2019; check.def file version of 30/07/2019

Datablock exp_1004 - ellipsoid plot

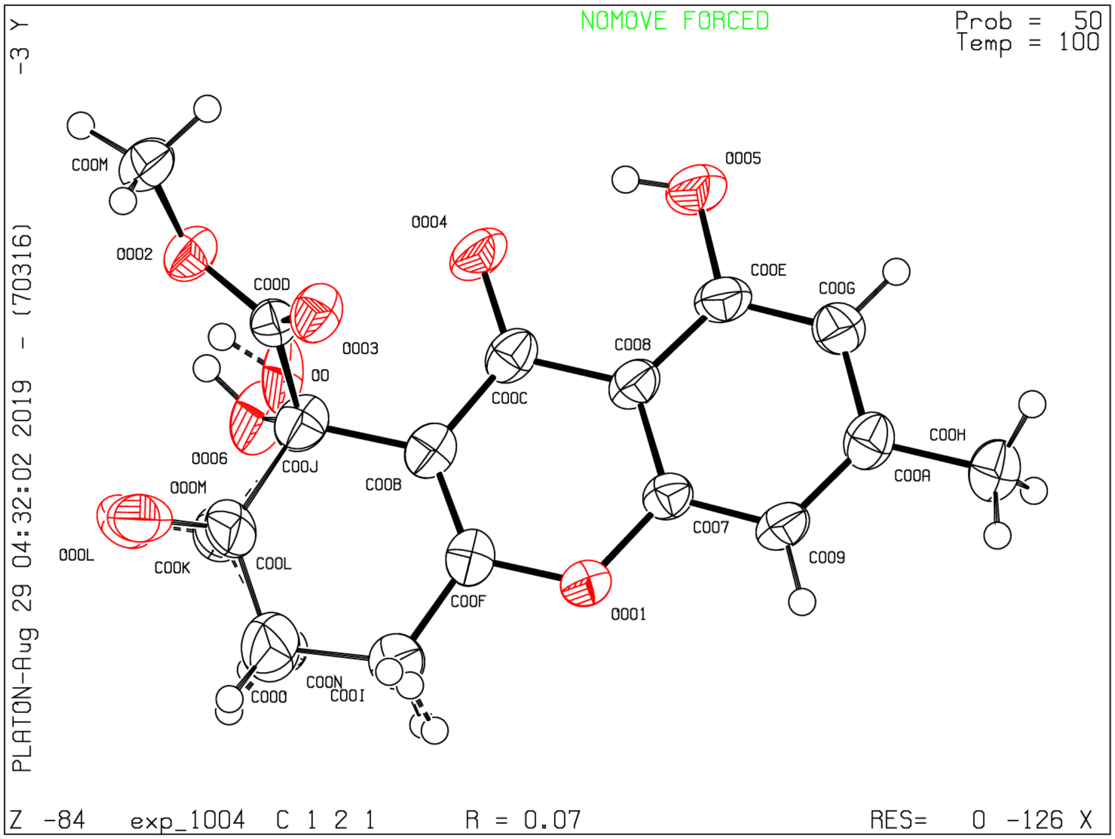

Supplement: File 2 — Crystal structure information files for 1, 3, and 5. [file Beilstein_J_Org_Chem-16-290-s002.zip › Crystal files/Checkcif1.pdf]
