# Supplementary material for: Absolute configurations of talaromycones A and B, α-diversonolic ester, and aspergillusone B from endophytic Talaromyces sp. ECN211
Source: Beilstein J Org Chem. 2020 Feb 28;16:290–6. doi: 10.3762/bjoc.16.28 (PMC7059540; doi:10.3762/bjoc.16.28)

checkCIF/PLATON report

You have not supplied any structure factors. As a result the full set of tests cannot be run.

THIS REPORT IS FOR GUIDANCE ONLY. IF USED AS PART OF A REVIEW PROCEDURE FOR PUBLICATION, IT SHOULD NOT REPLACE THE EXPERTISE OF AN EXPERIENCED CRYSTALLOGRAPHIC REFEREE.

No syntax errors found. CIF dictionary Interpreting this report

Datablock: 20190822_513

Bond precision: C-C = 0.0070 A Wavelength=1.54187

Cell:	a=7.3325(3)	b=13.9964(5)	c=9.0451(3)
	alpha=90	beta=111.569(8)	gamma=90
Temperature:	296 K		

	Calculated	Reported
Volume	863.28(7)	863.28(7)
Space group	P 21	P 1 21 1
Hall group	P 2yb	P 2yb
Moiety formula	C18 H20 O7	C18 H20 O7
Sum formula	C18 H20 O7	C18 H20 O7
Mr	348.34	348.35
Dx,g cm-3	1.340	1.340
Z	2	2
Mu (mm-1)	0.871	0.874
F000	368.0	368.0
F000’	369.30	
h,k,lmax	8,16,10	8,16,10
Nref	3146[1642]	2994
Tmin,Tmax	0.811,0.885	0.629,0.885
Tmin’	0.797	

Correction method= # Reported T Limits: Tmin=0.629 Tmax=0.885
AbsCorr = MULTI-SCAN

Data completeness= 1.82/0.95 Theta(max)= 67.982

R(reflections)= 0.0561(2566) wR2(reflections)= 0.1458(2994)

S = 1.084 Npar= 230

The following ALERTS were generated. Each ALERT has the format
test-name_ALERT_alert-type_alert-level.
Click on the hyperlinks for more details of the test.

Alert level C

```
PLAT089_ALERT_3_C Poor Data / Parameter Ratio (Zmax < 18) ........       7.14 Note
PLAT094_ALERT_2_C Ratio of Maximum / Minimum Residual Density ....       2.10 Report
PLAT234_ALERT_4_C Large Hirshfeld Difference C14      --C15      .       0.17 Ang.
PLAT340_ALERT_3_C Low Bond Precision on  C-C Bonds ...............      0.007 Ang.
```

Alert level G

```
CHEMS02_ALERT_1_G  Please check that you have entered the correct
            _publ_requested_category classification of your compound;
            FI or CI or EI for inorganic; FM or CM or EM for metal-organic;
            FO or CO or EO for organic.
            From the CIF: _publ_requested_category   CHOOSE FI FM FO CI CM CO or A
            From the CIF: _chemical_formula_sum :C18 H20 O7
PLAT398_ALERT_2_G Deviating  C-O-C    Angle From 120 for O3             107.1 Degree
PLAT398_ALERT_2_G Deviating  C-O-C    Angle From 120 for O6             109.6 Degree
PLAT791_ALERT_4_G Model has Chirality at C1         (Chiral SPGR)          S Verify
PLAT791_ALERT_4_G Model has Chirality at C7         (Chiral SPGR)          R Verify
PLAT791_ALERT_4_G Model has Chirality at C13        (Chiral SPGR)          R Verify
PLAT791_ALERT_4_G Model has Chirality at C16        (Chiral SPGR)          R Verify
```

---

0 **ALERT level A** = Most likely a serious problem - resolve or explain
0 **ALERT level B** = A potentially serious problem, consider carefully
4 **ALERT level C** = Check. Ensure it is not caused by an omission or oversight
7 **ALERT level G** = General information/check it is not something unexpected

1 ALERT type 1 CIF construction/syntax error, inconsistent or missing data
3 ALERT type 2 Indicator that the structure model may be wrong or deficient
2 ALERT type 3 Indicator that the structure quality may be low
5 ALERT type 4 Improvement, methodology, query or suggestion
0 ALERT type 5 Informative message, check

It is advisable to attempt to resolve as many as possible of the alerts in all categories. Often the minor alerts point to easily fixed oversights, errors and omissions in your CIF or refinement strategy, so attention to these fine details can be worthwhile. In order to resolve some of the more serious problems it may be necessary to carry out additional measurements or structure refinements. However, the purpose of your study may justify the reported deviations and the more serious of these should normally be commented upon in the discussion or experimental section of a paper or in the "special_details" fields of the CIF. checkCIF was carefully designed to identify outliers and unusual parameters, but every test has its limitations and alerts that are not important in a particular case may appear. Conversely, the absence of alerts does not guarantee there are no aspects of the results needing attention. It is up to the individual to critically assess their own results and, if necessary, seek expert advice.

Publication of your CIF in IUCr journals

A basic structural check has been run on your CIF. These basic checks will be run on all CIFs submitted for publication in IUCr journals (*Acta Crystallographica*, *Journal of Applied Crystallography*, *Journal of Synchrotron Radiation*); however, if you intend to submit to *Acta Crystallographica Section C* or *E* or *IUCrData*, you should make sure that full publication checks are run on the final version of your CIF prior to submission.

Publication of your CIF in other journals

Please refer to the *Notes for Authors* of the relevant journal for any special instructions relating to CIF submission.

PLATON version of 07/08/2019; check.def file version of 30/07/2019

Datablock 20190822_513 - ellipsoid plot

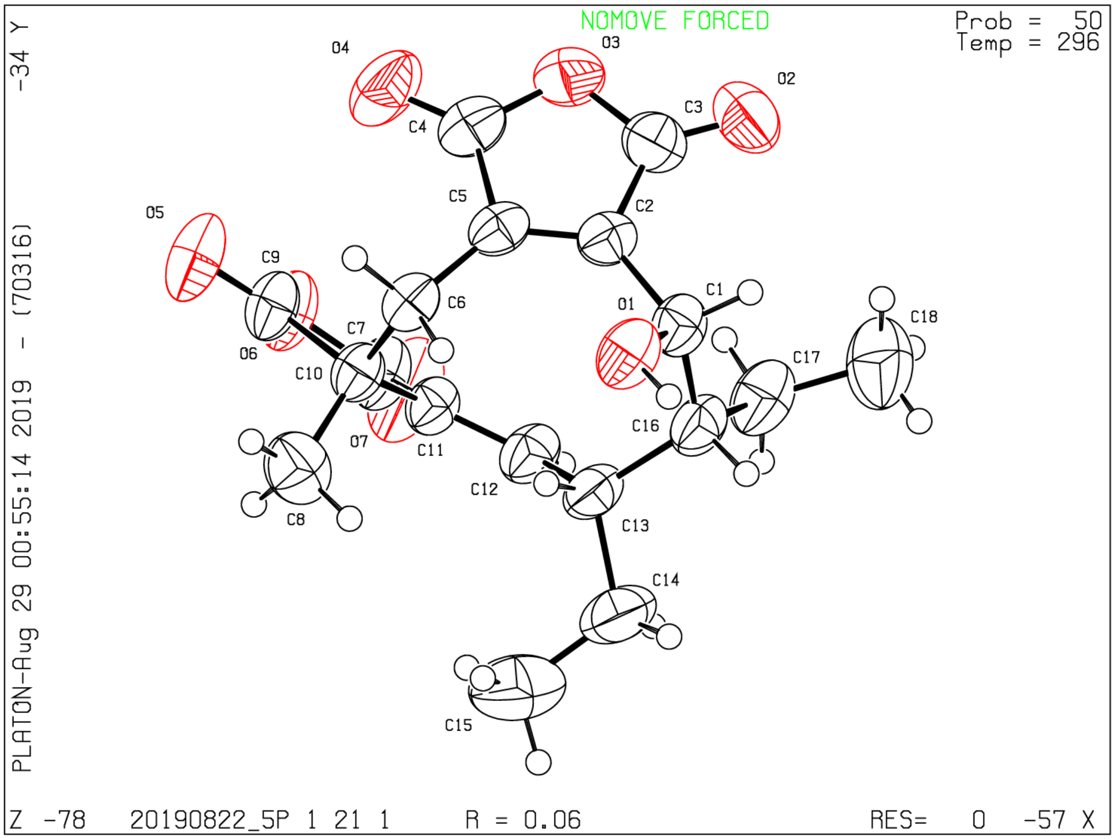

Supplement: File 2 — Crystal structure information files for 1, 3, and 5. [file Beilstein_J_Org_Chem-16-290-s002.zip › Crystal files/Checkcif5.pdf]
